# Human Papillomavirus in Head and Neck Cancer

**DOI:** 10.3390/cancers6031705

**Published:** 2014-08-18

**Authors:** Anna Rosa Garbuglia

**Affiliations:** Laboratory of Virology, “L. Spallanzani” National Institute for Infectious Diseases, Via Portuense, 292, 00149 Rome, Italy; E-Mail: argarbuglia@iol.it; Tel.: +39-06-5517-0692

**Keywords:** human papillomavirus, oral, cancer

## Abstract

Human papillomavirus (HPV) is currently considered to be a major etiologic factor, in addition to tobacco and alcohol, for oropharyngeal cancer (OPC) development. HPV positive OPCs are epidemiologically distinct from HPV negative ones, and are characterized by younger age at onset, male predominance, and strong association with sexual behaviors. HPV16 is the most prevalent types in oral cavity cancer (OCC), moreover the prevalence of beta, and gamma HPV types is higher than that of alpha HPV in oral cavity.

## 1. Introduction

During the last decade increasing attention has been paid to the presence of human papillomavirus (HPV) in the oral cavity and to its correlation with the development of oropharyngeal squamous cell carcinoma (OPSCC). In fact, recent epidemiological studies have shown a 225% increase in population-level incidence of HPV positive (HPV+) OPSCC between 1984 and 2004 [1], while HPV negative (HPV-) OPSCC decreased, probably due to the reduction of alcohol and tobacco consumption. This review provides an overview of the data available in the literature concerning HPV infection in the oropharynx, with particular attention to the prevalence of HPV by making a distinction between the alpha, beta and gamma HPV genera. In the last part a description of the methods for detection of beta, gamma, mu, nu HPV is provided to give useful information on the performance and characteristics of diagnostic systems used for the detection of genotypes belonging to these genera, which according to recent work would seem to be more prevalent than alpha HPVs in the oral cavity.

## 2. Papillomavirus: General Aspects, Evolution, and Classification

Papillomaviruses (PVs) are members of the genus *Papillomavirus*, and along with the genus *Polyomavirus*, they form in the family *Papovaviridae*. This is a family of epitheliotropic, non-enveloped viruses with a circular double stranded DNA genome. Papillomaviruses are predominantly species specific. In 1933 Shope linked the presence of cottontail rabbit papillomavirus (CRPV) to cutaneous papillomatosis in rabbits, and Peyton Rous studied the conversion of papilloma to squamous cell carcinoma in rabbits. Subsequent studies demonstrated that papillomaviruses infected all reptiles, birds and mammals [2]. More than 250 full-length genomes are sequenced and 148 HPV types are listed as Human papillomaviruses [3]. Overall, non-human PV has been identified to infect 54 different species belonging to 16 taxonomic orders, which include mammals, birds (parrot, chaffinch and francolin), reptiles (python and turtle). Anphibians do not represent a host for PV, and for this reason some believe that PV emerged 330 million years ago during the Carboniferous period, when the amniotes diverged from anamniotes. PVs are distributed worldwide in both humans and animals, but this wide distribution cannot be explained by an airborne transmission, as PVs can be transmitted only through close cutaneous contact with infected exfoliated skin cells or muco-mucosal contact. The main characteristic of PV is that it is a well-adapted biological behaviour in a specific host, with the possibility of latency and persistence; this feature suggests the hypothesis of co-evolution of PVs and their hosts as other pathogens. This hypothesis is supported by two pieces of evidence: the PV species are closed related in phylogenetic relatedness of their host and their evolution showed a synchronism with those of their hosts. Some examples E6 proteins of bovine (Bos *taurus*) PV type 1 (BPV1), European elk (Alces *alces*) PV (EEPV), mule, and deer (Odocoileus *virginianus* or *hemonius*) PV (DPV), all show their close phylogenetic relationships (for E6 protein function see below).

**Figure 1:**
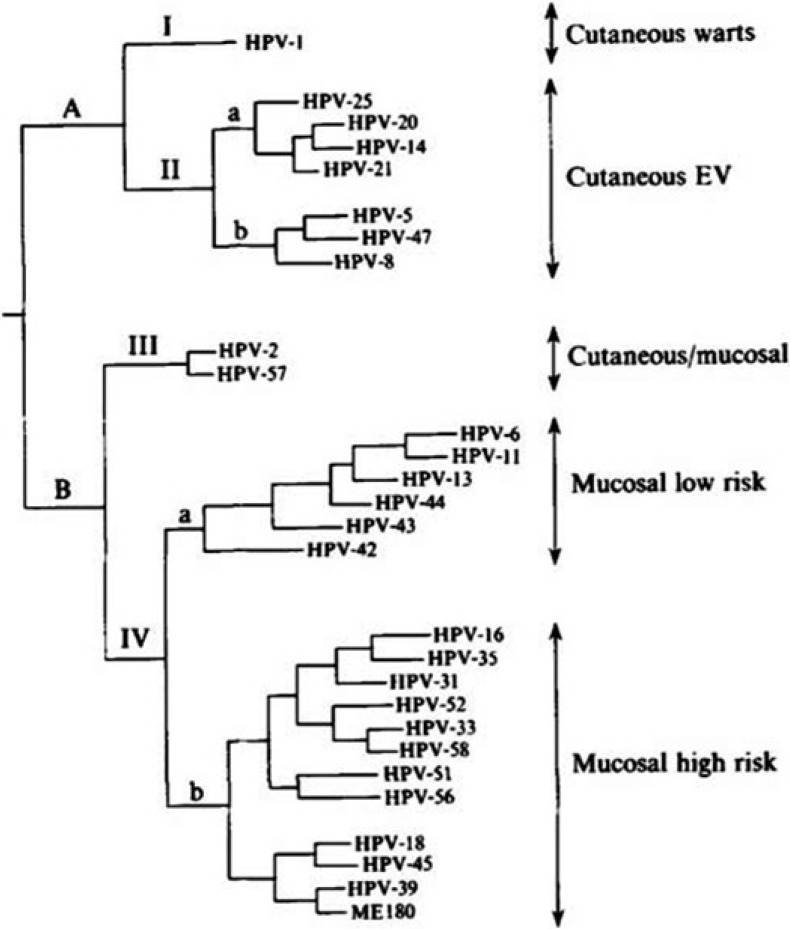
Phylogenetic tree constructed from the alignement of 384 nucleotides in the E6 genes of 28 HPVs using maximum parsimony analysis (PAUP 3.0). The labels on the right indicate the corresponding clinical category associated with each HPV type. (From Van Ranst *et al*. [4]).

In humans, using the parsimony phylogenetic method different HPVs are clustered into distinct groups corresponding to their tissue tropism and oncogenic potential. We could distinguish a first group (I) corresponding to HPV1, found in skin warts, and a second group considered to be EV (*epidermodyplasia verruciformis*) types. A second group (II) included mainly mucosal HPVs. A third group (III) with HPV2 and HPV57 was found to be associated to the skin, where they cause verruca vulgaris and in oral mucosa. The fourth group (IV) infect both genital and oral mucosa. It can be inferred that during the molecular evolutionary history of PVs, there was a clear division into PV which infected epithelial skin cells and PV which infected mucosal cells. It seems likely that the co-evolution occurred in an anatomic site specific manner (Figure 1) [4].

In absence of serological classification, HPVs have been classified according to their genotype. The HPV classification is made by analyzing nucleotide difference in a fragment of L1 gene, a gene present in all papillomaviruses. In fact the papillomavirus genome, which ranges from 6953 bp (*Chelonia mydas* papillomavirus genome type1, CmPV1) to 8607 bp (canine papillomavirus type1, CPV1), encodes different proteins, depending on the genera considered. All known papillomavirus encode five proteins: E1, E2 and E4, L1 and L2. L1 and L2 are structural, constituting the viral capsid, while E1 and E2 influence the replication cycle and transcription, E4 protein contributes to genome amplification efficiency and it favors high level expression of the viruses. In all papillomaviruses we also found a long control region (LCR) located between L1 and the beginning of the first ORF1 in the early region (E6 in the case of BPV1 and in the most human papillomaviruses). E5, E6, E7 proteins, having a key role in cellular growth regulation, immunosystem control, and in transformation process, are considered oncogenic.

The malignant transformation is prevalently associated with the activity of E6 and E7 proteins which promote cellular proliferation and prevent apoptosis [5]. The E6 and E7 proteins exercise their oncogenic activities by the interaction with tumour suppressor genes TP53 for E6 and retinoblastoma proteins for E7. Moreover High risk E6 reduces the surface expression of CDH1 naturally produced by epithelial cells during pathogen infection, reducing their capability to present human papilloma antigens [6]; the HR E6 and E7 also inhibit interferon synthesis through specific interaction with IRF-1 and IRF-3 [7,8].

Delta-papillomavirus, alpha-papillomavirus, kappa-paillomavirus contain E6, E7 and E5. Furthermore all fully-sequenced papillomaviruses contain at least one of these proteins. The L1 ORF is the most conserved gene within the genome and it is employed in PV phylogenetic analysis. The genera includes all PVs with a similarity ≥60% in L1 nucleotide sequences; species having a similarity encompass between 60% and 70%; types must have an identity between 70% and 89%. A new isolate is considered to be a new genotype if its L1 gene differs by more than 10% from the closest PV type. Differences between 2% and 10% identity define a subtype and less 2% a variant [9].

Recently new human HPV genotypes have been fully sequenced and with a metagenomic approach we could obtain more than 170 full genome HPV types [10]. They are subgrouped in five principal super groups. Genus Alpha (genital HPVs) contained 14 species (alpha1-14), genus Beta (EV group) contained five species (beta 1–5). In the Gamma genus (gamma 1–15) genotypes prevalently correlated with cutaneous lesions were found. The genus Mu includes HPV1, HPV 63, while genus Nu is only represented by HPV41.

Certainly it is very strange that the genus Mu and Nu are represented by two and one genotype respectively. Most likely with rolling circle amplification (RCA) method, metagenomics approach and new sets of primers able to amplify a larger number of genotypes [11] the number of genotypes belonging to these genera could rise rapidly.

## 3. HPV and Cancer

### 3.1. HPV Infection in Genital Sites

As described above the human papillomaviruses are distributed in five genera. Alpha genus includes mucosal and cutaneous HPV types. The cutaneous alpha types are considered as low risk and the most representative types are HPV2 and HPV 57, which cause common warts, and HPV3 and 10, which are correlated to flat warts [12,13]. Mucosal types, themselves, are classified in Low Risk (LR) and High Risk (HR). The HR are correlated with cervical and anal cancer development. HPV infections have been associated with 610,000 cases of genital cancers worldwide, of which the majority are cancers of the cervix, while 88% of related deaths occur in developing countries. The most common human papillomavirus types in cervical cancer are: HPV16 (57%), HPV18 (16%), HPV58 (5%), HPV33 (5%), HPV45 (5%), HPV31 (4%), HPV53 (3%), and HPV35 (2%) [14,15].

### 3.2. HPV and Head Neck Cancer

After exclusion of nasopharynx, head and neck squamous cell carcinoma (HNCCs) are responsible for approximately 550,000 new cases and 300,000 deaths in the world each year [16]. About three quarters of HNCCs occur in men, but substantial differences exist in the male-to female ratio across countries [17].

In general HNSCCs is largely attributed to environmental exposures. Tobacco use and alcohol consumption are the main risk factors. Tobacco represents the cause of 33% of HNCCs cases in North America, Latin America and Europe. Alcohol consumption causes 4% of HNSCCs. Other studies demonstrated that tobacco use and alcohol are related to 42% of HNSCCs deaths in low-income countries and 80% in high income countries [18]. Rates of oral cavity cancer have increased among men and women in some eastern and northern countries where tobacco use remains common, such as the Czech and Slovak Republics, Denmark, Estonia, and Finland. In addition, the rate of oral cavity cancer has increased among women only in a number of European nations, perhaps reflecting the delayed peak in smoking prevalence compared to men, as has been seen in other countries [19].

However, significant evidence suggests risk factors other than tobacco and alcohol could be involved in HNCCS development: (1) a small portion (15%–20%) of HNCCs occurs in non-smokers and non-drinkers; (2) the number of HNSCCs has increased even in developed countries where alcohol consumption and smoking have decreased. Oropharyngeal cancer (OPC) incidence has increased over the last 20 years in several countries, including Australia, Canada, Denmark, the Netherlands, Norway, United States, and United Kindom, where a decline in tobacco use has been observed [20].

Several authors have demonstrated that HPV presence in the oral cavity represents a risk factor for oral cavity or oropharyngeal cancer. Smith *et al*. found HPV to be a risk factor for oral cavity cancer (OCC) and OPC corresponding to 3.7 OR (95% confidence interval [XI] = 1.5–9.3), independent of alcohol and tobacco exposure [21]. Men with HPV infection acquired through sexual intercourse have a similar risk factor to develop OPC as that of women HPV+ to develop cervical cancer [22]. Gillison found a HPV prevalence of 22% when analyzing 253 biopsies of HNSCC specimens collected from June 1987 to October 1998. She employed MY09/MY11 PCR primers. HPV16 was present in 89% of specimens, HPV33 in three tumors, and one tumor harbored HPV16 and HPV31 [23]. Another study suggests that HPV in oropharyngeal cancer was found three time as often as in other HNSCC primary tumors (18.6% *versus* 6.1%; *p* < 0.002) [24]. Tonsillar carcinomas (a subset of oropharyngeal tumors) are positive in 60%–70% of cases in comparison to 6%–10% of tumors at other sites (*p* < 0.01) [25].

### 3.3. The Incidence of HPV-OPC

Studies based on cancer registries show a geographical correlation between the incidence of HNSCCs and cancer of the cervix and the penis. Furthermore, a significantly increased risk of HNSCC was reported subsequent to diagnosis of cervical or anal cancer [25]. HPV infection was detected in a broad range of benign head neck lesions, including juvenila laringeal papillomatosis, oral verrucal-papillary lesions, and squamous epithelial hyperplasia (heck’s disease). Cytological abnormalities, represented by koilocitosis, were observed in OPC HPV positive similarly to what was found in the genital tract [26].

Several studies show HPV specificity to tumor cell nuclei, high integration of HPV DNA into human genome, high HPV viral copy numbers and high level of expression of HPV oncogenes (E6 and E7) in tumors, all of which underscore a casual association of HPV with oropharyngeal cancers [27,28,29]. Sweden [30], Australia [31], and USA [1] are the three countries where an analysis concerning HPV HNSCCs incidence has been carried out. These studies show that the prevalence of HPV DNA in tumors, as well as incidence of HPV positive oropharyngeal cancers, has dramatically increased over time. For example in Sweden, HPV prevalence in tonsil cancers increased from 16% in the 1970s to 68% in the early 2000s, HPV associated-tonsillar cancer increased by 3.5-fold in women and 2.6-fold in men between 1970 and 2002 [32]. Likewise, in the USA, HPV prevalence in oropharyngeal tumors increased from 16% in the late 1980s to 72% in the early 2000s, whereas incidence of HPV negative OPC declined [1]. For these reasons, HPV is currently considered to be a major etiologic factor, in addition to tobacco and alcohol, for oropharyngeal cancer development. HPV positive OPCs are epidemiologically distinct from HPV negative ones, and are characterized by younger age at onset, male predominance, and strong association with sexual behaviors. Importantly HPV positive patients have substantially improved outcomes (28%–80% reductions in the risk of death), in comparison to HPV negative patients. The host genetic variation study of p53 gene polymorphisms evidenced a p53 sequence polymorphism encoding an arginine rather than a proline at position 72 favoring p53 degradation mediated by HPV in OSCCs [33]. Moreover a case-case study found that patients with HPV16 or HPV 18 DNA positive tumors compared to patients with HPV DNA negative OSCC did not show any difference in p53 arginine or p53 proline prevalence [34].

### 3.4. HPV Prevalence in the Oral Cavity

Despite the established relationship between HPV infection and HNSCC development, only a little data is available in the literature about HPV epidemiology in the oral cavity. A systematic review, which analysed oral HPV infections among healthy individuals took account of data published in 18 articles and related exclusively to alpha-papillomaviruses [35]. The analysis included 4,581 healthy individuals, 4.5% of which (95% CI: 3.9–5.1) showed positive for any HPV types, while 3.5% (95% CI: 3.0–4.1) harbored carcinogenic HPV, and 1.3% (95% CI: 1.0%–1.7%) were infected by HPV16. HPV16 represented 28.0% (51/182) of all infections detected in the oral region and 32.9% (51/155) of the infections were related to HR types. HR types considered in the study were: HPV18, 31, 35, 39, 52, 56, 58, and 66. HPV 16 was more prevalent in developing countries than in developed countries (4.3% *versus* 0.7%, respectively). A similar pattern was found for each HPV type analysed. Men and women had similar prevalence of detectable oral HPV (4.6% *versus* 4.4%, respectively).

Recently Gillison evaluated the prevalence of HPV in the oral cavity in people living in the US, checking the presence of 37 alpha-papillomaviruses among 5500 U.S. individuals (age: 14–69 years old). HPV was detected in 6.9% of analysed samples, HR HPV prevalence was 3.7% and LR-HPV represented 3.1% of positive samples; the prevalence of HPV16 was 1% [36]. The risk of HPV infection increased in HIV-positive patients. Beachler studied a group of 379 HIV-positive patients, with a median CD4T cell count of 524 cells/μL. HPV infection prevalence was 35% in women and 45% in men (mean 40%) in HIV-positive individuals, 21% was HR HPV type and 6.1% were HPV 16 [37]. The HR most frequently detected were HPV16, 59, and 33, those of LR were HPV55, 62, and 53. HIV-positive patients were 2.1 times more likely to harbour HPV in the oral cavity in comparison to HIV-negative people (ORs = 2.1; 95%: CI, 1.6–2.8, 40% *versus* 25% prevalence respectively). Lifetime oral sex behavior was associated with oral HPV prevalence in HIV-positive (*p* trend = 0.03), but not in HIV-negative (*p* = 0.11) people. The association between current CD4T cell count and oral HPV16 was significantly stronger than the association of current CD4 T cell count with all other HPV types (*p* = 0.02).

A study carried out in Italy on 166 HIV-positive MSM (men who have sex with men) reported a alpha HPV DNA prevalence of 20.1% at baseline a 21.3% after 6 months follow up: 75% (51/68) of individuals had persistent HPV infection after enrollment. HPV DNA detection was performed with MY09/MY11 and GP5+/GP6+ primers. Persistence rates of 71.4% with HR types and 76.7% in patients infected with LR type were observed. A negative relationship was found between HPV infection and CD4 T cell count [38]. A study carried out among 30 HIV-positive women in South Africa with a CD4 T cell count <300 prior to HAART introduction [39], reported a similar oral HPV prevalence (20%) suggesting that immune status impairment degree does not play a crucial role in oral HPV infection.

Another study carried out at the John Hopkins Hospital in Baltimore (MD, USA) among HIV-positive individuals—153 women (39%), 168 heterosexual men (43%), and 69 MSM (18%)—compared the α-HPV prevalence in oral and anal sites and their persistence. Baseline prevalence was significantly higher in anal sites than in oral sites (84% *versus* 28%, *p* < 0.001). Among oral HPV infections, 11% were multiple infections, and the main HPV types found in the oral cavity were: HPV55 (4.3%), 83 (4.1%), and 72 (3.8%). The HPV incidence in anal sites was significantly higher respect to the oral site (145 *vs*. 31 infections per 1000 person-months, HR = 4.7 95% CI: 3.6–6.2). In this study 38% of both anal and oral HPV infections were persistent (two consecutive HPV positive samples). After 6 months, 63% of anal HPV infections persisted against 46% of oral HPV infections. Age, tobacco use, and nadir CD4 T-cell count a baseline were not associated with anal or oral HPV persistence [40]. The high prevalence in anal site could be explained by the greater persistence of HPV in the ano-genital site. However, only a follow-up study can confirm this hypothesis.

In a HIM (HPV infection in men) survey carried out among men living in Mexico, Brazil and USA who were HIV-negative (*n* = 1876, aged 18–73 years), the incidence of oral infection was 4.4% and the median duration of oral HPV infection was 6.9 months (95% CI: 6.2–9.3) for all HPV types, 6.3 months (6.0–9.9) for oncogenic HPV, and 7.3 months (6.0-not estimable) for HPV16. Overall oral HPV infection cleared within 1 year, so HPV infection in healthy men seems not to be long lasting [41]. The HPV analysis was carried out by linear array (Roche Molecular Diagnostics, Alameda, CA, USA). Oral HPV16 (0.6%) was the most frequently acquired oncogenic HPV type, followed by types 59 and 39, which were detected in 0.5% and 0.3% of the enrolled individuals, respectively. HPV55 represented the most frequent non-oncogenic type, acquired in 2.0% of men. Among 115 men with an incidence of oral HPV infection, 15% (*n* = 17) HPV multiple infections were observed. No differences were observed in the risk of acquiring any HPV types across age groups (18–73 years old). Other variables (alcohol consumption, oral sex) were not significantly associated with oral HPV infection. These results are consistent with another study [42], whereby two other prospective studies, which investigated associations between oral sex and acquisition of oral HPV, reported different results. In one study performed on young men and women [43] no significant association between oral sex and incidence of oral HPV was observed, whereas another study that analysed oral HPV infection only in young men, showed that performing oral sex on a woman more than once per week was associated with an increased risk of acquiring oral HPV infection [44].

In Pickard’s study, where HPV oral prevalence was analyzed in young people (*n* = 1000, age: 18–30 years) HPV positivity was 2.4% (95% CI: 1.4–3.45) for all HPV types and HPV16 infection was lower than those reported in other studies: 0.2% (95% CI: 0–0.4).

In the HIM study the authors also calculated the HPV prevalence in oral sites [42]. HPV DNA prevalence in the oral region was 4.0% (95% CI 3.1% to 5%). Different prevalences were found in different countries: Brazil 2.1% (95% CI: 1.0–3.8), Mexico 5.9% (95% CI: 4.2–8.1), US 3.6% (95% CI: 2.3–5.4), *p* = 0.005. As reported in the incidence study, HPV 16 was the most frequently detected HR type (0.6%). HPV31, 35, 39, 52, 56, 58, and 59 were the other HRs found in the HIM cohort. HPV55 was the main LR type detected (1.1%). The prevalence of oral HPV increased non-significantly over increasing age categories. Only smoking-related variables were associated with a significant increase in the probability of detecting oral HPV infection (oral for current among: 2.5, 95% CI: 1.4–4.4).

In another study performed in Sweden on young people (408 women and 82 male oral HPV prevalence for alpha-papillomavirus 9.3% with no significantly difference between women and men (9.2% *vs*. 9.8% respectively). >2 HPV type were found in seven oral samples. HPV16 was the most prevalent HR type (2.9%, 95% CI: 1.7–4.8), other HR HPV types found were: HPV59 (1.4%, CI: 0.7–3.0), and HPV51 (1.2%, 95% CI: 0.6–2.7). HPV42 was the only low risk detected (1.0%, 95% CI: 0.4–2.4) [45].

Another Finnish study was carried out on pregnant women: 115/398 women acquired a HPV infection over six years of follow-up. HPV16 and HPV multiple infections represented the most frequently caused incidence of oral infections (65% and 12% respectively) and 46% of women cleared their HPV infection within 2 years. HPV58 had the highest clearance frequency (88.9%), while the other HR types have a mean clearance frequency of 33.3%. Mean clearance of HPV16 was 20.7 months, very similar to HPV multiple infection (23.5 months). HPV6 was the main LR detected and showed the longest persistence (mean 4.6 months) with a clearance frequency of 25% [46].

Another recent study in Brazil reported an oral alpha-HPV prevalence of 1.3%. The samples were analysed by PCR for E6 and E7 genes specific for HPV 6, 11, 16, 18, 31, 33, 45, and with a second set of primers MGMY09/11 [47].

On the contrary, a second study carried out in Brazil reported an oral HPV prevalence of 23.2% among 125 healthy individual using MY09/MY11 primers [48]. The different HPV 16 prevalences reported in these studies could be explained by different factors such as: (1) age of patients enrolled, (2) their immune status; (3) sexual behavior or (4) their paricipation in the HPV screening programme for ano-genital cancer prevention. The heterogenicity of the population studies do not allow any reliable conclusions.

### 3.5. Βeta, Gamma and other Cutaneous HPV Types in Oral Sites

Genera beta, gamma, mu, and nu are considered to be correlated with benign cutaneous lesions and only occasionally have they been found in skin cancers. Some beta types however can be associated with the development of neoplastic disease (Bowen’s disease, actinic keratosis) [49].

As previously described the beta papillomavirus genus have been divided into five species with related HPV types (beta-1: HPV5, 8, 12, 14, 19, 20, 21, 24, 25, 36, 47, 93; beta-2: HPV 9, 15, 17, 22, 23, 37, 38, 80; beta-3: HPV49, 75, 76; beta-4: HPV92 and beta-5:HPV96). Gamma papillomaviruses have been divided into five species (gamma-1: HPV4, 65, 95; gamma-2: HPV48, gamma-3: HPV50, gamma-4: HPV60; gamma-5: HPV88) [9]. Additionally, species alpha-2, 4, 8 contain 13 HPV types which are associated with cutaneous lesions (HPV2, 3, 7, 10, 25, 28, 29, 40, 43, 57, 78, 91, 94) [9].

Cutaneous HPV types represent a risk factor for SCC (skin cell carcinoma) and basal cell carcinoma (BCC), particularly in immunocompromised patients. In general alpha cutaneous HPV types are rarely detected in both SCC and BCC, whereas HPV types belonging to beta1, beta 2, and beta 3 species are the most frequently detected in SCC compared to controls. Among beta 4 species only HPV92 is elevated in SCC in comparison to controls [50]. Only recently has the presence of cutaneous HPV in the oral cavity been considered, and the results are very interesting and highlight a greater cutaneous HPV prevalence in comparison to those of mucosal HPVs. Rare and non conclusive are results for about genus gamma, mu, nu in SCC development.

The first work which gave information about Beta-papillomavirus and Gamma-papillomavirus prevalence in oral cavity was Bottalico’s paper [51]. The analysis was carried out with FAP primers able to detect 46 HPV types (see page 788, [51]). He compared alpha, beta, gamma HPV prevalence in oral rinse to cervical swabs.

Among HIV-positive patients with HPV positive oral rinse (*n* = 35) 21 samples (60%) were infected with at least one alpha type, 20 samples (57%) with at least one beta type, and seven samples (20%) with at least one gamma type. There was no statistically significant association between CD4 T cell count and HPV positivity. Among HIV-negative individuals with HPV DNA in oral rinse (*n* = 117), 29 samples (25%) harbored alpha HPV types, 74% (*n* = 87) were infected with beta HPV types, and 14 samples (12%) with gamma-HPV types. HPV5 (*n* = 17) was the most prevalent beta HPV type. In HPV DNA positive cervical specimens (*n* = 257), 96% were positive for alpha HPV types (*n* = 248), 2% (*n* = 6) were positive for beta HPV types, and beta HPV types were detected in nine specimens (4%). Overall eight novel beta HPV types and 12 novel gamma HPV types were identified. Among the beta genus, HPV120 and 124 were most frequently observed, whereas HPV121 and HPV134, belonging to the gamma genus, were each found twice.

In another study 52 HIV-positive patients were enrolled in a study to assess HPV DNA prevalence in oral rinse. HPV DNA was detected in 45 patients (87%), 25% of them had HPV multiple infections. Αlpha HR HPV was detected in 12 individuals 23%, and HPV58 and 68 were the most frequently found. Among alpha LR types HPV32 and 42 (8%) were the most common types. In 46% of individuals beta or gamma HPV types were detected: HPV8 was the most frequent beta type and 101/103 were the most commonly found gamma types. All cases with detectable HIV RNA viral load were positive for HPV DNA. Patients on HAART were also most frequently positive for beta or gamma HPV types [52].

The detection of HR and LR alpha-10 types (HPV6, 11) was elevated among people with a history of genital warts, while individuals with a history of cutaneous warts were more likely to harbour beta or gamma HPV types (37% *versus* 43%; *p* = 0.397). This is the highest prevalence of beta and gamma genus in the oral cavity.

This result can be explained in part by the state of immunosuppression of the subjects, which provokes an attenuation of local immunity and in part also potential chronic perondontitis, but only studies with a larger number of subjects and for those in which a past history of risk factors such as oral leukoplakia, periontitis or tonsillar inflammation is known, may this high HPV prevalence in the oral cavity perhaps sometimes be explained.

Different beta and gamma papillomaviruses prevalence was found in oral samples and nasal samples of volunteer Danish healthcare staff [53]. HPV DNA was detected by MGP primers [54] and with FAP primers [55]. HPV DNA was detected in 6% of oral samples and 50% of the nasal samples. There was difference in HPV prevalence among different age groups; 6% of nasal samples harboured HPV multiple infections. Alpha-papillomavirus, beta-papillomavirus and gamma-papillomavirus were observed in 0.96%, 3.8%, and 16% of oral samples respectively, and 2.6%, 31%, and 16% of nasal samples, respectively. Beta 1 was the most representative HPV in nasal specimens (17%). For the nasal samples, the most frequently HPV types detected were: HPV76 (4.6%), HPV124 (3.9%), HPV24 (3.2%) and HPV134 (2.9%). Notably FA167, a subtype of HPV76, was detected in seven samples. Novel gamma HPV types FA9, FA45, FA54 were found in several nasal samples.

In another study [56] oral rinses were collected from 7466 women from Costa Rica, and HPV DNA was detected with a Line probe assay (LiPA_25_ Labo Biomedical Products, Rijswijk, The Netherlands), which employs the SPF_10_ primer set, with a Diassay BV assay [57] and with a broad-spectrum PCR multiplex genotyping assay [58]. The alpha mucosal HPV type was 1.9% (95% CI: 1.4–2.4) and 55 HPV infections were detected. The most common HR were HPV16 (0.4%, 95% CI: 0.2–0.7), HPV51 (0.3%, 95% CI: 0.2–0.6), and HPV52 (0.2%; 95% CI: 0.1–0.4). LR were represented prominently by HPV66 (0.2%; 95% CI: 0.1–0.4) and HPV44 (80.2%; 95% CI: 0.1–0.4). Overall prevalence of beta HPV types was 18.6% (95% CI: 15.3–22.3). The most common beta types were HPV24 (3.8%, 95% CI: 2.3–5.9), HPV38 (3.0%, 95% CI: 1.7–4.9), and HPV5 (2.2%, 95% CI: 1.1–3.9). Alpha, gamma, mu, nu cutaneous HPV types were rarely present in this specimen collection, so the authors regrouped these genera and their combined prevalence was 4.5 (95% CI: 1.1–23.9). The most common type detected was HPV1 (1.4%, 95% CI: 0.6–2.9). Single cutaneous HPVs were detected in 15.3% (95% CI: 12.2–18.7) of analysed specimens, whereas multiple cutaneous infections represented 6.4% (95% CI: 4.4–8.9) of positive samples. No significant association were found with chronic diseases of tonsils, adenoids, sinusitis and oral HPV infections. Moreover, when the authors considered a subset of women (*n* = 246) detection of alpha HPV increased the odds detection of beta cutaneous HPV within the oral region (OR, 4.8, 95% CI: 1.2–20.0), though cutaneous HPV types were 10 times more common in oral region than mucosal HPV types (21.6%). Unlike the mucosal HPV types, HPV cutaneous infection was not correlated to sexual activity.

Paolini and colleagues, employing MY09/MY11 primers, GP5+/GP6+, CP65/CP70, FAP59/FAP64 primers observed a different prevalence of cutaneous types (without specifying alpha, beta, or gamma) in different groups. In healthy individuals group cutaneous HPV types were 25% *versus* 8% mucosal HPV types. Among people with non-malignant lesions, cutaneous HPV prevalence was 51% *versus* 12.7% mucosal HPV types, whereas in group of samples from oral cancer cutaneous HPV type prevalence was 20.5% *versus* 21.5% HPV mucosal HPV types. Multiple infections represented 4.6% of all infections, and cutaneous and mucosal co-infection was found only in one sample. Beta HPV types represented 64% out the total HPV types. Moreover no viral transcript RNA was found in any of the positive samples for cutaneous HPV DNA suggesting a non-involvement of cutaneous HPV DNA in tumour development in this anatomical site [59] (See Table 1).

However, the prevalence of new genotypes in the oral cavity might increase with the introduction of new HPV search methods such as RCA or metagenomics. In a recent paper DNA enrichment by RCA was used and this approach allowed the detection of four new genotypes in the oral rinse of healthy individuals [11]. All four genotypes belonged to the genus gamma papillomavirus. Phylogenetic analysis showed that these genotypes clustered in different clades: HPV171 showed high similarity to HPV169, HPV172 was mostly closely related to HPV156; HPV73 was most closely related to HPV4 and OSL37 was most closely related to HPV114 (69%) similarity.

## 4. HPV in Oral Site and Biomarkers

Unlike HPV in cervical cancers, where the secondary prevention by screening prevention and HPV detection led to a decline in cervical cancer incidence, in oropharyngeal squamous cell carcinoma (OSCC) no associations between HPV presence and cytological abnormalities were found.

Fakhry and colleagues carried out a cross sectional population study (PAP1), in which cytology specimens were collected from patients presenting with abnormalities. In a nested case-control study (PAP2) bilateral tonsillar cytology samples were collected at 12 months intervals from HIV-infected individuals. HPV16 infection was not associated with atypical squamous cells of unknown significance (ASCUS), but HPV16 was associated with OSCC among individuals with accessible oropharyngeal lesions (HPV16 was detected in squamous cell carcinoma in oropharyngeal and tonsillar cytology specimens [60].

**Table 1:** Main papers about beta, gamma HPV type prevalence description in oral cavity.

Authors, Years [Ref]	Subject Type	Sample Type	% Alpha Types ^	%Beta Types ^	% Gamma Types
Bottalico *et al*., 2011 [51]	HIV positive (*n* = 52)	Oral rinse (HPV +, *n* = 35)	60 ^	57 ^	20 ^
	HIV negative (*n* = 317)	Oral rinse (HPV+ *n* = 117)	25 ^	74 ^	12 ^
	HIV negative women, (*n* = 1,807)	Cervical sample (HPV+, *n* = 14)	96	2	4
Fatahzadeh *et al*., 2013 [52]	HIV positive (*n* = 52)	Oral lavage sample (HPV+ *n* = 45)	23	46	§
Forslund *et al.*, 2013 [53]	HIV negative (*n* = 312)	Oral samples(*n* = 311, 6% HPV+)	0.96	31	1.6
		Nasal samples (*n* = 304, 50% HPV+)	3	31	23
Lang Kuhs *et al.*, 2013 [56]	Women, control arm (*n* = 2926)	Oral and gargle rinse	1.9	18.6 (93/500 ^#^)	4.0 (20/500 ^#^)
	Vaccine arm (*n* = 2912)	Oral and gargle rinse	1.6	18.6 (93/500 ^#^)	4.0 (20/500 ^#^)
Paolini *et al.*, 2013 [59]	A:healthy patients *n* = 25	Oral rinse and mouth swabs	8	25 °	
	B: non malignant lesions (*n* = 47)	Oral rinse and mouth swabs	12.7	51 °	
	C: cancers (*n* = 78)	Biopsies from neoplastic lesions	21.8	20.5 °	

HIV: Human immunodeficiency virus; HPV+: human papillomavirus positive; n, number. ^: Samples with HPV infection from >1genus are counted for each genus; §: The authors did not distinguish HPV positivity between beta and gamma types. ^#^: Beta, gamma, mu, nu papillomaviruses are detetected in a sub-group of 500 women, and the results from both arm were pooled, since vaccination status did not affect detection of cutaneous HPV types. °: Beta and gamma positivity has been pooled.

Perhaps the evaluation of the persistence of HPV, particularly of HR, may be associated with the onset of OSCC, but studies in this direction must be conducted. Several studies have attempted to assess biomarkers associated with HPV OSCC correlation. For instance, Slebos [61] analyzed the oral epithelium of 10 HPV-negative and 10 HPV-negative OPC, and 10 normal adults using a standardized global proteomic analysis platform. This system allowed the analysis of 2653 proteins. In HPV-positive OPC there was a high expression of argininosuccinate synthase1 (ASS1) indicating that in HPV-positive OPC cellular methabolism needs a higher amount of the essential amino acid arginine. Furthermore the transcription factors E2F1 and E2F4 are expressed higher in HPV-positive OPC with a novel method based on a mass spectrometry (MS)-based detection method. The E2F-family member involvement suggests a probable pRB inactivation by E7 protein.

Two additional up-regulated transcription factors were found: upstream transcription factor1 (USF1) and C/EBP homologous protein (CHOP). USF1 is a basic helix-loop-helix (HLH) transcription factor and regulates genes involved in cellular differentiation. CHOP is involved in the apoptosis regulation process. In HPV-positive OPC a lower level of immunoglubulins, lactotransferrins were observed. Many of the hallmark differences identified by the studies relate to direct action of the viral oncogenes E6 and E7. In addition to E2F1 and EF4 proteins, a higher level of MYC and MAX response protein was found HPV-positive OPC. MAX is a bHLH-LZ protein required for MYC activity and through their association, the MYC/MAX complexes are able to activate the transcription of MYC target genes. It has been shown that higher MYC levels can be detected in the presence of E6. MYC is a key transcriptional factor involved in cell cycle progression, proliferation, metabolism, transformation and apoptosis. In the presence of E6, MYC also induces TERT transcription, through its binding to the TERT promoter proximal E-box [62,63].

These very promising results are still preliminary. In fact they have been conducted on a limited number of samples and the predictive power of changes of factors such as MYC/MAX or E2F1, an alteration of this transcript profile, has not yet been established.

In another study comparing 50 HPV positive head neck carcinoma to 50 HPV negative HNSCC, the authors identified two differently expressed proteins: prostate stem cell antigen (PSCA), which was upregulated, and eukaryotic elongation factor 1 alpha (EEF1α), which was down regulated in HPV-positive cells [64].

PSCA was discovered 15 years ago and has been found in prostate, bladder, cancer, ovarian and renal carcinomas. To date only one study reported decreased a PSCA expression (100-fold) in HNSCC [65]. EEF1α is a GTP-binding protein that interacts with aminoactil-tRNA to recruit amino acids and it has a key role in the elongation phase of protein translation. In another study different expression of genes such as SYCP2, which is involved in nuclear structure and meiosis, CRABP2, an important factor in cell differentiation, RFC5, which has a repair DNA activity, ZNF238, a transcription regulation factor and KLK8, a protein involved in epidermidis development was associated to HPV+ HNSCC [66]. Evaluating these overall data it is observed that different studies show the presence of several differentially expressed proteins in the HPV-positive HNSCC tumors. Perhaps this may be due to the different tumors considered (tonsil, pharynx, oral cavity). Overall these findings lead us to conclude that there are still no established biomarkers associated with HPV + HNC cancers with predictive value.

Furthermore being that these data refer to a small number of tumor samples and that they have not been confirmed by studies carried out on a substantial number of HPV positive oral samples (rinses, swabs, gargles), they still cannot be used in clinical practice or to decide prevention measures.

## 5. Methods for Detecting Alpha, Beta, Gamma, mu, nu Papilloma Viruses

HPV PCR testing for beta, gamma, mu, and nu papillomaviruses has been developed to detect HPV in human skin and some of these sets of primers have been employed in HPV detection in oral sites [36,51]. Most of the HPV detection methods use general primers for the L1 open reading frame. The “FAP” PCR system represents the most frequent method used for detecting beta, gamma, and some alpha HPV viruses in the oral cavity [55]. With this set of primers no amplification could be obtained for types 1, 2, 35, 41, 44, 55, 63, 66, 71 or 74. HPV40 and HPV58 give bands different from those expected: 700 bp and 260 bp, respectively. FAP sequences generated by primers FAP59 and FAP 64 (478 bp, HPV 8 type) constitute about 30% of L1 region, and they could be employed for the identification of HPV types [67]. The detection limit of FAP PCR was calculated for some HPV types only (HPV50, 20, 30) and ranged between 1 and 10 copies/reaction. FP59/FAP64 are able to detect PVs infecting various animals such as chimpanzees, gorilla, elk, cow [68].

To improve the sensitivity of this PCR system, a nested internal primer pair was introduced to a single FAP59/64 single round system following the “hanging droplet” approach. FAP 6085F and FAP6319 R corresponded to nucleotides 6085-6104 and 6319-6296 of the HPV8 genome, yielding a nested amplicon of 235 bp. The analytical sensitivity of nested PCR is about 10-fold compared to that of single round PCR [69]. Moreover, FAP nested PCR may preferentially detect beta-1 types. Furthermore another study demonstrated that the FAP6085 and FAP64 primer combination (FAP6085/64) was more sensitive than other primer sets in single round assays [70].

SKF1 and SKR1 (I Round), SKF2/SKR2 (II Round) primer couples were employed in a nested PCR by Sagasawa to detect the main cutaneous HPVs [71]. It was confirmed that L1 genes of HPV types 1a, 2a, 3, 4, 7, 10, 27, 28, 29, 57, 60, 63, and 65 were efficiently amplified by these new sets of primers. Conversely beta papillomavirus HPV5, 8, 47, 92, 93, 96 were not amplified. The sensitivity of this method was about 7000–9000 copies of HPV DNA/reaction. The low sensitivity and the limited HPV type number detected probably has discouraged the use of these primers in beta, gamma HPV detection in oral sites.

Berkhout and colleagues used instead a nested PCR to amplify all epidermodyslpasia verruciformis (EV) HPV types. This method consists in of a first-step PCR amplification carried out with CP65 and CP70 primers able to amplify HPV5, 8, 9 12, 14a, 15, 17, 19, 20, 21, 22, 23, 25, 34, 36, 37, 38, 41, 46, 48, 49, and 50. In the second step CP66 and CP69 primers were used in second round PCR. These sets of primers have different capability to amplify different genotypes. The two step PCR succeed in detecting mucosal HPVs, including HPV6, 11, 13, 16, 18, 31, and 33.This method was not able to amplify HPV3, 4, 10, whereas some types including HPV2, 26, 27 were efficiently amplified only by the first round PCR [72]. This author improved HPV3, 10 detection employing other primer sets, which allowed the detection of 16 putative novel EV-associated HPV types [73]. The sensitivity of CP66/CP69 method ranged from 5 to 50,000 copies/reaction [74].

Harwood and colleagues employed degenerate primers in a nested PCR. They employed in a first round a set of primers (F14-B15) for the L1 region as already described by Shamanin [75]. In the second round the PCR product of amplification of the first step was used as a template employing a different set of primers for specific species: CN3F-CN3R, group alpha (HPV3, 10, 28, 29, 77); CN2F-CN2R, group alpha 4 (HPV 2, 27, 57); C4F-C4R for beta 2 ( HPV4, 4850, 60, 65) in order to improve the sensitivity obtained with F14-B5. This method allowed detection of 0.05 EV HPV copies per cells [76].

One PCR system is based on amplification of a conserved E1 region, and is able to amplify 25 beta types. The PCR products are employed in a reverse hybridization system (RHA) containing 23 genotypes specific probes, while other probes are used in pattern recognition of genotypes. HPV5, 9, 12, 14, 15, 17, 19, 23, 24, 25, 36, 37, 38, 49, 75, 76, 80, 92, 93, 96, 8, 21, 20, 22, 47 could be identified with this system [57]. The PCR set of primer described by Harwood *et al*. [76] were employed to produced biotynilated amplimers that were used for HPV typing with Luminex 1001 S system (Luminex Corporation, Austin, TX, USA). This method allows detection and typing of the following genotypes: alpha-genera (HPV2, 3, 7, 10, 27, 28, 29, 40, 43, 57, 77, 91, and 94), gamma-genus (HPV4, 65, 95, 48, 50, 60, and 88), mu-genus (HPV1, 63), and nu-genus (HPV41). The analytical sensitivity of HSL-PCR/MGP ranges from 1 to 10 copies per PCR HPV types 1 and 2, from 10 to 100 copies per PCR HPV4, 7, 10, 43, 48, 63, and 95; from 100 to 1000 copies per PCR for HPV types 3, 27, 29, 57, 60, 65, and 77; and from 1000 to 10,000 copies per PCR for HPV types 28, 40, 41, 50, 88, 91 and 94 [58].

BGC-PCR/RLB is a method based on HPV PCR followed by reverse-line blotting (BGC-PCR/RLB) able to detect 25 beta-PV, and the analytical sensitivity ranges from 10 copies (HPV 75, 80, 92, 93, and 96) to 100 copies (HPV76) [77].

Another typing assay consisting of biotinylated PCR products of the nested FAP method [69] has been employed. These are hybridized to type-specific probes coupled to fluorescence labelled polystyrene beads and analyzed using Luminex technology. This genotyping assay, called “multiplex cutaneous papillomavirus genotyping assay” (McPG) , is able to detect 58 cutaneous HPVs of beta-genus (5, 8, 12, 14, 19, 20, 21, 24, 25, 36, 47, 93, 9, 15, 17, 22, 23, 37, 38, 80, 107, 49, 75, 76, 86), gamma-genus (41, 65, 95, 48, 50, 60, 88, 101, 103), mu-genus (1, 63), and nu-genus [78]. Recently a comparison of three different methods, BGC-PCR/RLB, multiplex cutaneous papillomavirus genotyping (McPG) and FAP PCR was carried out [79]. The agreement between the three methods was 73%. This weak concordance could be explained by different analytical sensitivity for the different HPV genotypes intrinsic to each method. The main beta, gamma, mu, nu detection PCR methods are described in Table 2.

The FAP PCR system is very useful in identifying new genotypes, it shows a good sensitivity for almost all types detected, but it loses its sensitivity in HPV multiple infection detection. The other two methods showed a more limited range number of type detection, but they more efficiently identify HPV multiple infections. Concerning alpha HPV detection one suggests other exhaustive reviews [80,81].

## 6. Conclusions

The change of epithelial status from normal, cellular atypia, dysplasia, carcinoma *in situ* to invasive carcinoma, as demonstrated in cervical cancer, could not be identified in oral-oropharyngeal cancer, so it is difficult to define a prevention measure.

A recommendation would be to repeat the HPV DNA test after 6, 12 months, when the HPV DNA test is positive at baseline, especially if a high-risk HPV was found, or if the subject has a cervical or anal dysplasia or if he has immune compromised status. Regarding beta and gamma genotypes, the data about their persistence and correlation with the onset of OPC tumors are lacking. Their high prevalence in oral rinses suggests a high tropism of these HPV types for oral mucosa. Though they are not considered high risk, they can promote benign neoplasia, but only follow-up studies aimed to closely monitor HPV presence in the oral cavity could establish beta-gamma HPVs capacity to influence OPC development.

**Table 2:** Main PCR and primers used in beta, gamma, mu, nu HPV types detection.

Primer Name	Sequence (5' → 3')	HPV Region	Product Length (bp)	Genotypes Detected	References
SKF1	GAGCAAAATTTCCAACAAAGG	L1	210–238		[68]
SKR1	ATACCATAGAYCCACTRGG				
SKF2	AAATATCCTGATTATTTRGGMATG				
SKR2	AAACYATAGAGCCACTWGG			1a, 2a, 3; 4; **6**; 7; 10; **11**; **16**; **18**, 27; 28; 29, 57; 60; 63; 65	
FAP59	TAACWGTIGGICAYCCWTATT	L1	478		[55]
FAP64	CCWATATCWVHCATITCICCATC			65 genotypes. No band was detected for HPV types: 1; 2; 35; 41; 44; 55; 63; 66; 71; 74	
FAP6085	CCWGATCCHAATMRRTTTGC	L1(nested of FAP59/64 PCR)	235		[69]
FAP6319	ACATTTGIAITTGTTTDGGRTCAA			≈10 fold greater sensitivity compared to that of single round PCR [55]. No band was detected for HPV types: 1; 2; 35; 41; 44; 55; 63; 66; 71; 74	
PM-A	ACTGACCAAAGCTGGAAATC	E1	117		[57]
PM-B	TCTTGCAGAGCATTGAAACG			5; 8, 9; 12; 14; 15; 17; 19; 20; 21; 22; 23; 24; 25; 36; 37; 38; 47; 49; 75; 76; 80; 92; 93; 96	
CP65	CA(A/G)GGTCA(C/T)AA(C/T)AATGG(C/T)AT	L1	452–457		[72]
CP70	AA(C/T) TTTCGTCC(C/T)A(A/G)AG (A/G)A(A/T) ATTG(A/G)TC			5b; 8;9; 14a; 15; 17; 19; 20; 21; 24; 25; 34; 36; 38; 41; 48; 49; 50	
CP66	AATCA(A/G)(A/C)TGTTT(A/G)TTAC(A/T)GT		389	3, 5b; 8;9; 10,14a; 15; 17; 19; 20; 21; 24; 25; 34; 36; 38; 41; 48; 49; 50	[73]
CP69	G(A/T)TAGATCC(A/T)ACAT(C/T)CCA(A/G)AA				

Alpha genotypes detected by these methods are indicated in bold.

The presence of beta and gamma genotypes in the oral cavity have been weakly investigated. The recent studies by Martin with a metagenomic approach have highlighted the presence of new genotypes of HPV, of which their involvement in the onset of tumors in OPC remains entirely unknown. Of course, the biggest gap is the absence of a system for detection of a larger number of beta and gamma HPV genotypes, including the latest identified and untyped ones. More effort should be applied to improve multiple infection system detection, because this factor (multiple infection) could also promote the persistence of HPV in the oropharynx.

The presence of cutaneous HPV and their influence in OPC is still unclear. Paolini *et al*. [59] do not observe any viral transcript in non-malignant lesion or cancer, so excluding an active role in neoplastic transformation of these genotypes, but this fact cannot explain the persistence of these genotypes in the oral cavity for at least 6 months, as described in other studies. Work on the persistence of the cutaneous human papillomavirus and the standardization of the detection of viral transcripts could resolve this doubt.
